# Abdominoplasty Combined with Wide Abdominal Plication and Hip Expansion By Fat Grafting: Addressing the Umbilicus.

**DOI:** 10.1093/asjof/ojaf018.012

**Published:** 2025-05-13

**Authors:** Tara Mather, John LoGiudice, Wilberto Cortes

**Affiliations:** Medical College of Wisconsin, Milwaukee, WI; Medical College of Wisconsin, Milwaukee, WI; Medical College of Wisconsin, Private Practice, The Woodlands, TX

## Abstract

**Goals/Purpose:**

The umbilicus is considered the central aesthetic unit of the abdomen and holds great significance in most cultural beauty standards. An abdominoplasty with wide abdominal plication (from external oblique to external oblique) frequently performed by the senior author (W.C.) creates challenges in forming a new umbilicus. The location and shape of the new umbilicus at the abdominal flap can be distorted from the vertical vector forces on the abdominal flap when it is closed under tension and the horizontal vector forces that result from wide abdominal fascial plication and medial migration of the abdominal flap. The authors describe an umbilicoplasty that counteracts the forces of distortion in the abdominoplasty with wide plication. The technique involves controlling the shape, tension of the closure, and location of the new umbilicus using four permanent sutures that connect the abdominal fascia to the superficial fascia at the new umbilical site. They can be adjusted to improve the new umbilicus's depth, size, and shape.

**Methods/Technique:**

A retrospective chart review using medical records and CosmetAssure claims from February 2014 to October 2023 of patients undergoing abdominoplasty by the senior author (W.C.) was performed. Pre-procedure photos were captured, along with follow-up images at one, three, six to eight months, and, when available, at the one-year mark.

**Results/Complications:**

1,172 patients underwent umbilicoplasty in the context of abdominoplasty with wide plication, all performed by the senior author (W.C.). The average age was 38 (range 20 to 65), and the average BMI was 28 (range 18 to 42). Complications included delayed wound healing and/or abnormal scarring resulting in constricted umbilicus (10, 0.8%) and infection (6, 0.5%). There were no umbilical losses.

**Conclusion:**

This umbilicoplasty technique effectively creates a natural-looking umbilicus in abdominoplasty with wide abdominal plication. The low complication rates indicate that it is a safe technique that yields aesthetically pleasing results. It is a promising procedure that might be integral to any abdominoplasty technique or abdominal reconstruction procedure.

